# Facteurs prédictifs de succès des étudiants en première année de médecine à l'université de Parakou

**DOI:** 10.11604/pamj.2016.23.87.8527

**Published:** 2016-03-15

**Authors:** Thierry Adoukonou, Francis Tognon-Tchegnonsi, Emile Mensah, Alexandre Allodé, Jean-Marie Adovoekpe, Prosper Gandaho, Simon Akpona

**Affiliations:** 1 Faculté de Médecine, Université de Parakou, Parakou, Benin

**Keywords:** Academic performance, medical student, predictors, Academic performance, medical student, predictors

## Abstract

**Introduction:**

Plusieurs facteurs dont les notes obtenues au BAC peuvent influencer les performances académiques des étudiants en première année de médecine. L'objectif de cette étude était d’évaluer la relation entre les résultats des étudiants au BAC et le succès en première année de médecine.

**Méthodes:**

Nous avons réalisé une étude analytique ayant inclus l'ensemble des étudiants régulièrement inscrits en première année à la Faculté de Médecine de l'université de Parakou durant l'année académique 2010-2011. Les données concernant les notes par discipline et mention obtenue au BAC ont été collectées. Une analyse multivariée utilisant la régression logistique et la régression linéaire multiple a permis d’établir les meilleurs prédicteurs du succès et de la moyenne de l’étudiant en fin d'année. Le logiciel SPSS version 17.0 a été utilisé pour l'analyse des données et un p<0,05 a été considéré comme statistiquement significatif.

**Résultats:**

Parmi les 414 étudiants régulièrement inscrits les données de 407 ont pu être exploitées. Ils étaient âgés de 15 à 31 ans; 262 (64,4%) étaient de sexe masculin. 98 étaient admis avec un taux de succès de 23,7%. Le sexe masculin, la note obtenue en mathématiques, en sciences physiques, la moyenne au BAC et la mention étaient associés au succès en fin d'année mais en analyse multivariée seule une note en sciences physiques > 15/20 était associée au succès (OR: 2,8 [1,32- 6,00]). Pour la moyenne générale obtenue en fin d'année seule une mention bien obtenue au BAC était associée (coefficient de l'erreur standard: 0,130 Bêta =0,370 et p=0,00001).

**Conclusion:**

Les meilleurs prédicateurs du succès en première année étaient une bonne moyenne en sciences physiques au BAC et une mention bien. La prise en compte de ces éléments dans le recrutement des étudiants en première année pourrait améliorer les résultats académiques.

## Abstract (en)

**Introduction:**

Several factors including grades obtained in the Baccalaureate can influence academic performance of first year medical students. The aim of this study was to evaluate the relationship between results achieved by students taking Baccalaureate exam and student academic success during the first year of medical school.

**Methods:**

We conducted an analytical study that included the whole number of students regularly enrolled in their first year of medical school at the university of Parakou in the academic year 2010-2011. Data for the scores for each academic discipline and distinction obtained in the Baccalaureate were collected. Multivariate analysis using logistic regression and multiple linear regression made it possible to determine the best predictors of success and grade point average obtained by students at the end of the year. SPSS Statistics 17.0 was used to analyse data and a p value p < 0.05 was considered significant.

**Results:**

Among the 414 students regularly enrolled, we could exploit the data on 407 students. They were aged 15-31 years; 262 (64.4%) were male. 98 were enrolled with a success rate of 23.7%. Concerning men, the scores obtained in mathematics, in physical sciences, the grade point average obtained in the Baccalaureate and honors obtained in the Baccalaureate were associated with their success at the end of the year, but in multivariate analysis only a score in physical sciences > 15/20 was associated with success (OR: 2,8 [1,32-6,00]). Concerning the general average grade obtained at the end of the year, only an honor obtained in the Baccalaureate was associated (standard error of the correlation coefficient: 0,130 Beta =0,370 and p=0,00001).

**Conclusion:**

The best predictors of student academic success during the first year were a good grade point average in physical sciences during the Baccalaureate and an honor obtained in the Baccalaureate The inclusion of these elements in the enrollement of first-year students could improve academic performance.

## Introduction

Les premières écoles de médecines en Afrique ont ouvert leurs portes au début du 20^ème^ siècle avec un accroissement de leur nombre avec les indépendances et surtout une nouvelle croissance de ce nombre dans les années 90 [1]. Depuis l'année académique 2000-2001 l'université de Parakou a ouvert ses portes avec une entité de formation de médecine. Ceci répond à un besoin de former des médecins pouvant comble le déficit existant. D'ailleurs la pénurie en médecin en Afrique est criard [2] et au Bénin il existe un médecin pour [3]. Ainsi faute de médecins la plupart des centres de santé du système sanitaire béninois au niveau périphérique sont tenus et gérés par des non-médecins en accord avec un triste constat fait dans toute l'Afrique au sud du Sahara [4]. D'abord appelée Ecole de médecine elle est devenue une faculté de médecine et a déjà mis sur le marché de l'emploi 142 médecins. Toutefois il est à noter que depuis le taux d’échec ne cesse d'augmenter avec parallèlement une croissance démographique dans les différentes années de formation. Aussi faut-il remarquer que le taux de succès à la première session en première année est quasi nulle et devrait interpeller. Il est important de souligner que le nouveau système de recrutement dans les entités de formation, initialement prévu pour assurer un égal accès aux formations professionnalisées doit être réévalué. De tout ce qui précède il nous paraît judicieux d’étudier ces résultats et surtout de voir si les critères actuels de recrutement pouvaient expliquer ces taux d’échec ou plus simplement les facteurs associés ou expliquant ces résultats en première année. L'objectif de cette étude était d’étudier les chances de succès des étudiants en première année de médecine selon les critères de recrutement et les performances de ces étudiants au BAC.

## Méthodes

**Cadre d’étude:** La faculté de médecine de l'université de Parakou a servi de cadre à cette étude. Le recrutement en première année s'effectue sur étude de dossiers et tenant compte des notes obtenues au BAC et de la mention de l’étudiant.

### Méthodes d’étude

**Type d’étude:** Il s'est agi d'une étude observationnelle, transversale à visée descriptive et analytique

**Période d’étude:** Cette étude s'est déroulée du 1^er^ Octobre au 29 Décembre 2011

**Population d’étude:** Elle est constituée de l'ensemble des étudiants régulièrement inscrits en première année de médecine à la Faculté de médecine de l'université de Parakou au titre de l'année académique 2010-2011.

**Echantillonnage:** Nous avons réalisé un échantillonnage non probabiliste exhaustif incluant donc l'ensemble des étudiants.

**Critère de jugement:** La variable principale à l’étude et principal critère de jugement était le succès de l’étudiant en fin d'année c′est-à-dire à l'issue de la deuxième session des examens académiques.

**Outils et méthode de collecte des données:** Nous avons à partir d'une base de données conçues, validée et testée nous avons collecté les informations relatives aux variables à l’étude. Les données étaient recherchées dans les bases de données de la scolarité de la faculté et de l'université et parfois complétées en interrogeant d'autres sources chaque fois que cela était nécessaire. Ces données étaient complétées au fur à mesure que nous avons les résultats académiques.

**Variables:** Variable dépendante: c'est le critère de jugement tel que défini ci-dessus. Le critère secondaire de jugement était la moyenne obtenue par l’étudiant à la fin de la deuxième session. Variables indépendantes. Elles sont socio démographiques (âge, sexe), et concernent: les informations relatives aux critères de recrutement: notes dans les différentes disciplines au BAC, la mention au BAC, moyenne obtenue au BAC, mode de recrutement (boursier, non boursier); les informations relatives aux différentes disciplines enseignées: note obtenue dans chaque discipline, système de cours (enseignant présent ou missionnaire).

**Traitement et analyse des données:** Les données collectées avec les logiciels Excel 2000, étaient traitées et analysées avec le logiciel SPSS 16.0. Les variables qualitatives étaient exprimées en pourcentage et les variables quantitatives en moyenne avec un écart-type. Le critère de jugement principal qui est une variable dichotomique est exprimé en pourcentage. Le critère de jugement secondaire qui est une variable quantitative est exprimé en moyenne avec un écart-type. Pour l'analyse univariée concernant le critère de jugement principal les comparaisons de fréquences étaient effectuées avec le test de chi-2 ou le test exact de Ficher selon le cas et celle des moyennes par le test de Student. En analyse multivariée concernant le critère principal de jugement le modèle de régression logistique fut utilisé avec des itérations successives pas à pas descendante en introduisant dans le modèle initial simultanément toutes les variables significatives en analyse univariée avec un seuil de sortie des résultats fixé à 0,05. La force et l'intensité de l'association était estimées par l'odds-ratio avec son intervalle de confiance à 95%. Pour le critère de jugement secondaire l'analyse multivariée était effectuée en utilisant une régression linéaire multiple avec détermination des meilleurs prédicteurs de la moyenne de l’étudiant en fin d'année.

## Résultats

**Description de la population d’étude:** Sur un total de 414 étudiants inscrits au titre de l'année académique seules les données de 407 étudiants étaient exploitables et avaient donc pu être analysées soit un taux de réponse ou de complétude de 98,31%. Parmi les 407 étudiants inclus dans cette étude 262 étaient de sexe masculin soit 64,4%. Le sex-ratio était de 1,81. Ils étaient âgés de 15 à 31 ans avec une moyenne de 19,97ans (+/-1,78 ans); la médiane étant de 20 ans.

**Fréquence de réussite en fin d'année:** Parmi les 407 dont les dossiers étaient complétés 91 étaient reçus en fin d'année soit une fréquence de 22,4%. Mais rapporté au total des 414 inscrits et des 98 admis le taux était de 23,7%.

**Données relatives aux critères de recrutement:** Le Tableau 1: résume les données relatives aux moyennes obtenues par discipline au BAC en français, mathématiques, sciences physiques et biologie. En terme de mention obtenue au BAC 43,7% des étudiants avaient obtenu la mention passable, 33,4% la mention assez-bien, 21,9% la mention bien et 1% la mention très-bien. Les étudiants admis en année supérieure étaient âgés en moyenne de 19,89 (+/-1,57) ans et ceux ayant connu un échec de 19,99 (+/-1,84) ans mais la différence n’était pas significative (p=0,686). Parmi les 262 sujets de sexe masculin 70 étaient reçus soit un taux de 26,7% et parmi les 145 filles 21 étaient reçues soit un taux de 14,5%, la différence entre ces taux est statistiquement significative (p=0,005). Le Tableau 2 résume les données relatives aux moyennes obtenues par discipline au BAC et le succès en première année. La répartition des étudiants suivant les mentions obtenues au BAC et le succès en fin d'année est résumée dans le Tableau 3.

**Tableau 1:** Données relatives aux notes obtenues par discipline au BAC. Parakou, 2011

Disciplines	Moyenne	Médiane	Ecart-type	[Min-Max]
Français	8,87	9,0	2,59	[1-19]
Biologie	15,44	16,5	2,92	[7-19]
Mathématiques	9,98	10,0	3,65	[1-19]
Sciences Physiques	11,38	12,0	3,09	[4-18]
Moyenne générale	11,72	11,26	1,92	[9,0-16,16]

**Tableau 2:** Comparaison des étudiants suivant les notes obtenues au BAC et suivant le statut reçu ou échec. Parakou, 2011

	ReçuMoyenne (+/-Ecart-type)	Echec Moyenne (+/-Ecart-type)	P
Français	9,46 (3,23)	8,79 (2,51)	0,386
Biologie	16,69 (2,46)	15,27 (1,84)	0,098
Mathématiques	12,31 (3,45)	9,67 (3,58)	0,014
Sciences Physiques	13,92 (2,46)	11,04 (3,02)	0,001
Moyenne au BAC	13,31 (1,84)	11,51 (1,84)	0,001

**Tableau 3:** La répartition des étudiants suivant les mentions obtenues au BAC. Parakou, 2011

	Reçu N (%)	Echec N (%)	OR [IC95%]	P
Passable	18 (10,1)	160 (89,9	1	0,0001
Assez-bien	44 (32,4)	92 (67,8)	4,25 [2,3-7,8]	
Bien	27 (30,3)	62 (69,7)	3,9 [2,0-7,5]	
Très-bien	2 (50)	2 (50)	8,9 [1,2-67,0]	

OR: odds-ratio, IC95%: intervalle de confiance à 95%

**Analyse multivariée utilisant le modèle de régression logistique:** En analyse multivariée après introduction dans le modèle des variables sexe, note en mathématiques, sciences physiques, mention et moyenne obtenue au BAC seule la note obtenue en sciences physiques au BAC était associée au succès en fin d'année. Ces données sont résumées dans le Tableau 4.

**Tableau 4:** Facteur prédictif du succès en première année de médecine, analyse multivariée. Parakou, 2011

	OR [IC95%]	p
Note sur 20 en physique au BAC (>15/<10)	2,8 [1,32-6,00]	0,0075

**Facteurs associés à la moyenne générale (critère de jugement secondaire) de l’étudiant en fin d'année:** En analyse univariée étaient associés à une forte moyenne générale le sexe (p=0,0001), la moyenne obtenue au BAC (p=0,0001), la note obtenue en mathématiques (p=0,012), la note obtenue en sciences physiques (p=0,001) et la mention obtenue au BAC (p=0,00001). La note obtenue en biologie au BAC n’était pas associée à la moyenne générale de l’étudiant en fin d'année (p=0,067). En analyse multivariée utilisant le modèle de régression linéaire multiple seule la mention obtenue au BAC était associée à la moyenne générale de l’étudiant. L’équation finale du modèle s’écrit comme indiquée dans le Tableau 5. En conclusion les meilleurs prédicteurs de la réussite en première année étaient la note obtenue en sciences physiques et la mention obtenue au BAC.

**Tableau 5:** Modèle final de prédicteurs de succès en première année de médecine; Parakou, 2011

	Coefficient de l'erreur standard	Bêta	p
Constante	0,257		
Mention au BAC	0,130	0,370	0,00001

## Discussion

Dans cette étude il s'est agi d’évaluer les résultats académiques des étudiants en première année de médecine à l'université de Parakou durant l'année académique 2010-2011. Pour ce faire nous avons réalisé une étude analytique reprenant les principaux déterminants du recrutement des étudiants. Ainsi 23,7% des étudiants inscrits durant l'année académique avaient connu un succès et passaient en deuxième année du premier cycle d’études médicales (PCEM2). Les meilleurs prédicteurs du succès en PCEM2 étaient la note obtenue au BAC en sciences physiques, la moyenne générale et la mention obtenue. Par ailleurs en analysant les moyennes des étudiants dans les disciplines de la terminale on s'aperçoit que seule la note obtenue en biologie était importante. Ceci pourrait s'expliquer par le fait que tous les étudiants provenaient de la série D (sciences de la vie et de la terre) et le reste de la série C (Mathématiques et sciences physiques). Cette étude a permis de prédire les chances de succès des étudiants en première suivant leurs performances au BAC. Ainsi la probabilité de réussir est plus importante pour un étudiant de sexe masculin ayant obtenu une moyenne supérieure à 15 sur 20 en sciences physiques et la mention bien au BAC. Toutefois il faut tenir compte de l'horizon temporel et spatial de cette étude. En effet on ne peut généraliser les résultats de cette étude à l'ensemble des facultés de médecine ni à toutes les années académiques. Plusieurs autres facteurs non pris en compte dans cette étude pourraient aussi influencer les résultats académiques. Par exemple le système d'enseignement, la présence ou non d'enseignant sur place, les conditions d’étude, l'accès des étudiants à une documentation’ La faculté de médecine de Parakou est une jeune faculté ne disposant pas encore d'enseignants suffisants dans toutes les disciplines fondamentales telles la biophysique, la biologie humaine, l'anatomie, la physiologie. Tous ces enseignements se faisant par mission d'enseignement les cours sont regroupés avec parfois 40 heures de cours de la même discipline dans une même semaine et ce parfois à quelques semaines de l’évaluation de l’étudiant qui n’était pas habitué à ce système. D'autres facteurs tels les difficultés financières des étudiants (beaucoup d’étudiants sont sans bourse et soutien financier), le lieu de résidence des parents de l’étudiant et la psychologie et les motivations de l’étudiant de même que les stratégies d'apprentissage [5].

Tous ces facteurs pouvaient constituer une limite à cette étude. Cette dernière n'a nullement l'intention de produire un modèle unique de prédiction de succès d’étudiant en PCEM1. La motivation première de l’étude étant de voir si les critères actuels de recrutement en première année basés uniquement sur les performances des étudiants au BAC ne pourraient pas expliquer en partie le taux d’échec obtenu comparé à la faculté de médecine de Cotonou ou ce taux d’échec est inférieur à 20%. Toutefois les résultats obtenus à Parakou sont meilleurs par rapport à ceux obtenus par exemple à la faculté de médecine de la Pitié-Salpêtrière à Paris en France. En effet sur quatre ans (1997-2001) 376 étudiants sont admis en PCEM2 sur un total de 2635 soit un taux de 14,3% [6]. Dans cette étude les auteurs ont retrouvé aussi des résultats superposables aux nôtres quant aux prédicteurs de succès. Ainsi les chances de succès varient suivant la mention: 2 à 6% pour les étudiants sans mention ou avec mention passable, de 15 à 20% pour les mentions Assez-Bien, de 38 à 52% pour les mentions Bien, et de 53 à 67% pour les mentions Très-Bien. Il est à souligner que dans cette Faculté il s'agit d'un concours de passage en PCEM2 et que tous les étudiants en première année s'inscrivent sans sélection préalable. Une autre différence observée dans cette étude est la forte proportion d’étudiantes. Ces facteurs identifiés ne sont pas les seuls prédicteurs. En effet dans une étude visant à évaluer les facteurs influençant les performances académiques des étudiants en médecine en Inde les auteurs ont retrouvé que le sexe masculin, l′incapacité à surmonter le précédent échec à la première tentative, la difficulté de compréhension des enseignements, la dépression auto-évaluée, les troubles du sommeil et la pression reçue des parents et des pairs et, le manque de motivation dans le choix de la carrière ont été significativement associée à une mauvaise performance [7]. Ces auteurs n'ont pas retrouvé de relation entre le statut socioéconomique, l'absentéisme et les performances académiques. Pour les étudiants eux-mêmes très peu d’études ont été consacrées à leurs perceptions sur les facteurs pouvant influencer leurs résultats académiques. Une récente étude conduite auprès d’étudiants en médecine à Londres [8] a montré que pour les étudiants, les facteurs qui pourraient influencer leurs performances seraient l'engagement dans l'apprentissage, les méthodes d'apprentissage, l'intérêt de l'application de ce qu'ils apprennent dans leur pratique future ainsi que la socialisation de l’étudiant (qualité de la relation avec les autres étudiants).

## Conclusion

En conclusion cette étude, loin d'identifier tous les facteurs expliquant l’échec en première année de médecine, a permis au moins de définir assez clairement les critères objectifs de recrutement en première années. Mais il faudra entreprendre une étude plus large prenant en compte l'ensemble des facteurs potentiels et en tenir compte globalement pour améliorer les résultats académiques en première année en faculté de médecine de la jeune université de Parakou.

### Etat des connaissance sur le sujet

- La probabilité de succès des études médicales est fortement influencée par les performances des étudiants au BAC.

### Contribution de notre étude a la connaissance

- Plus que les performances des étudiants c'est les notes obtenues dans les disciplines scientifiques (Sciences physiques chimiques et technologiques) et la mention qui influencent le succès en première année d’études médicales.

## References

[1] Mullan F, Frehywot S, Omaswa F, Buch E, Chen C, Greysen SR (2011). Medical Schools in sub-Saharan Africa. Lancet..

[2] WHO (2006). Working together for health; the world health report 2006.

[3] (2006). Annuaire des statistiques sanitaires 2006. SNIGS Ministère de la Santé.

[4] Mullan F, Frehywot S (2007). Non physician clinicians in 47 sub-Saharan African countries. Lancet..

[5] Stegers-Jager KM, Cohen-Schotanus J, Themmen AP (2012). Motivation, learning strategies, participation and medical school performance. Med Educ..

[6] Catala et Galietti Analyse des critères de réussite du PCEM1 au Chu Pitié-Salpêtrière.

[7] Mandal A, Ghosh A, Sengupta G, Bera T, Das N, Mukherjee S (2012). Factors affecting the performance of undergraduate medical students: a perspective. Indian J Community Med..

[8] Todres M, Tsimtsiou Z, Sidhu K, Stephenson A, Jones R (2012). Medical students' perceptions of the factors influencing their academic performance: an exploratory interview study with high-achieving and re-sitting medical students. Med Teach..

