# Erratum: facteurs prédictifs de succès des étudiants en première année de médecine à l’université de Parakou

**DOI:** 10.11604/pamj.2016.24.249.10102

**Published:** 2016-07-18

**Authors:** The Pan African Medical Journal

**Affiliations:** 1 The Pan African Medical Journal, Kampala, Uganda

**Keywords:** Erratum, academic performance, medical student, predictors, Erratum, academic performance, medical student, predictors

## Abstract

Cet erratum corrige l’article: “Facteurs prédictifs de succès des étudiants en première année de médecine à l’université de Parakou”. The Pan African Medical Journal. 2016;23:87. doi:10.11604/pamj.2016.23.87.8527

## Abstract (en)

This erratum corrects article: “Erratum: predictors of success among first-year medical students at the university of Parakou”. The Pan African Medical Journal. 2016;23:87. doi:10.11604/pamj.2016.23.87.8527

## Erratum

Dans la version originale de ce manuscrit [1] le tableau 5 a été omis. Cela a été corrigé à la fois dans la version PDF et HTML dudit manuscrit.
